# Enrichment of cancer stem cells via β-catenin contributing to the tumorigenesis of hepatocellular carcinoma

**DOI:** 10.1186/s12885-018-4683-0

**Published:** 2018-08-03

**Authors:** Harshul Pandit, Yan Li, Xuanyi Li, Weizhong Zhang, Suping Li, Robert C. G. Martin

**Affiliations:** 1 Division of Surgical Oncology, Hiram C. Polk Jr. M.D. Department of Surgery, University of Louisville School of Medicine, 511 S Floyd ST MDR Bldg Rm326A, Louisville, KY 40202 USA; 2 Department of Hand Surgery, China-Japan Union Hospital, Jilin University, Changchun, Jilin, 130022 China; 3 Department of Pharmacology and Toxicology, University of Louisville School of Medicine, Louisville, KY 40202 USA; 4 Division of Surgical Oncology, Department of Surgery, University of Louisville School of Medicine, 315 E. Broadway - #312, Louisville, KY 40202 USA

**Keywords:** Hepatocellular carcinoma, Wnt/β-catenin signaling, Cancer stem cells, Epithelial cell adhesion molecule (EpCAM), Tumor initiating cells

## Abstract

**Background:**

Hepatocellular carcinoma (HCC) is among the deadliest cancers due to its heterogeneity, contributing to chemoresistance and recurrence. Cancer stem-like cells (CSCs) are suggested to play an important role in HCC tumorigenesis. This study investigates the role of Wnt/β-catenin pathway in CSC enrichment and the capabilities of these CSCs in tumor initiation in orthotopic immunocompetent mouse model.

**Methods:**

HCC-CSCs were enriched using established serum-free culture method. Wnt/β-catenin pathway activation and its components were analyzed by western blot and qRT-PCR. The role of β-catenin in enrichment of CSC spheroids was confirmed using siRNA interference. Tumorigenic capabilities were confirmed using orthotopic immunocompetent mouse model by injecting 2 × 10^6^ Hepa1–6 CSC spheroids or control cells in upper left liver lobe.

**Results:**

The serum-free cultured Hepa1–6 cells demonstrated self-renewal, spheroid formation, higher EpCAM expression, increased Hoechst-33342 efflux, and upregulated Wnt/β-catenin signaling. Wnt/β-catenin pathway upregulation was implicated with the downstream targets, i.e., c-MYC, Cyclin-D1, and LEF1. Also, we found that GSK-3β serine-9 phosphorylation increased in Hepa1–6 spheroids. Silencing β-catenin by siRNA reversed spheroid formation phenotype. Mice injected with Hepa1–6 CSC spheroids showed aggressive tumor initiation and growth compared with mice injected with control cells.

**Conclusions:**

Successfully induced Hepa1–6 spheroids were identified with CSC-like properties. Aberrant β-catenin upregulation mediated by GSK-3β was observed in the Hepa1–6 spheroids. The β-catenin mediated CSC enrichment in the induced spheroids possesses the capability of tumor initiation in immunocompetent mice. Our study suggests plausible cell dedifferentiation mediated by β-catenin contributes to CSC-initiated HCC tumor growth in vivo.

**Electronic supplementary material:**

The online version of this article (10.1186/s12885-018-4683-0) contains supplementary material, which is available to authorized users.

## Background

Hepatocellular carcinoma (HCC) is the fifth most common cancer in men and the seventh in women worldwide, and is the third major cause of cancer-related deaths [1, 2]. HCC is often diagnosed at advanced stage when patients cannot be qualified for potentially curative treatment modalities, such as liver resection and liver transplantation. These patients are only left with options for palliative treatments such as chemotherapy, radiotherapy, drug-loaded beads, ablation, and Sorafenib. Most HCC patients’ 5-year relative survival rate is 7% and they show disease recurrence with advance-stage intrahepatic metastases [3, 4]. Evidence suggests that cancer stem cells (CSCs), a poorly differentiated subpopulation of cancer cells within the tumor microenvironment, contribute to aggressive tumor progression, chemoresistance, and recurrence in HCC patients [5].

The CSC model proposes a hierarchical population in the tumor microenvironment, where apex CSCs are the least-differentiated subpopulation retaining self-renewal capability with asymmetric division and having the highest tumorigenic potential. Subsequently differentiated cancer cells in the hierarchy lose tumorigenic potential in decremental order, ending with terminal cancer cells with little to no tumorigenic potential [6, 7]. The CSC tumor model has been proven to demonstrate clinical relevance in primary HCC, chemoresistance and recurrent HCC [5, 8, 9]. Based on tumorigenic potential and stemness characteristics, many studies have identified CSCs from human HCC tissues and HCC cell lines expressing different stem cell markers: EpCAM+, CD90+, CD44+, CD133+, AFP+, OV6+, and ALDH1+ [5, 9–12]. These diverse markers of CSCs have been thought to be a result of heterogeneity of CSCs, and no single marker can define the CSCs exclusively [13]. In HCC, EpCAM emerged as an important CSC surface marker and EpCAM+ cells correlate with worse prognosis and possess CSC-like properties showing tumor-initiating capabilities with as few as 200 cells in a nude mouse model [11, 14–17]. EpCAM is a target of Wnt/β-catenin signaling, and inhibiting Wnt/β-catenin signaling has been shown to destroy EpCAM+ cells [16, 18].

The canonical Wnt/β-catenin signaling is considered as fundamental pathway in stem-cell biology which regulates several cellular events including cell proliferation [19]. In the absence of Wnt ligand, β-catenin forms a complex with APC, Axin, and GSK-3β (destruction complex), and is phosphorylated at S33/S37/T41 positions and causes cytoplasmic β-catenin to undergo ubiquitin-mediated proteaosomal degradation. Upon binding of Wnt ligand to frizzled receptor (Fz) and a member of the LDL receptor family Lrp5/6 on cell membrane, active non-phosphorylated GSK-3β gets phosphorylated at the ser9 position and turned inactive; this leads to uncoupling of β-catenin from the destruction complex. The stabilized β-catenin translocates into the nucleus, where it activates target genes by binding to TCF/LET transcription factors [20]. Two of the most reported CSC markers in HCC, EpCAM and CD44, have been identified as the transcription targets of the canonical Wnt/β-catenin pathway [17, 21, 22]. Although studies have reported the role of Wnt/β-catenin signaling in the self-renewal and maintenance of CSCs in HCC [23, 24], the role of Wnt/β-catenin signaling in HCC-CSCs is largely unknown. Also, tumorigenic potential of these HCC-CSCs in immunocompetent orthotopic mouse model was not studied.

In this study, we investigated the role of Wnt/β-catenin in HCC-CSCs spheroids generation and maintenance. Our study for the first time successfully induced spheroids in regular tissue-culture plates with an adherence environment, providing a spontaneous spheroid formation which is closely related to the in vivo condition. The function of HCC-CSCs spheroids in tumor formation was further studied in an immunocompetent orthotopic HCC mouse model, for the very first time.

## Methods

Routine methods, antibody catalog numbers (Additional file 1: Tables S1 and S2), and additional regent details (Additional file 1: Table S3) are provided in Additional file 1.

### Cell lines used, cell culture, and CSCs enrichment in serum-free condition

Hepatoma cell lines, Hepa1–6 (CRL-1830), Hep3B (HB-8064) and HepG2 (HB-8065), were obtained from American type culture collection (Manassas, USA). All cell lines are validated every 6 months or obtained a new ATCC stock every 6 months. All experiments were performed between 5th and 20th passage. Hepa1–6 cells were grown in DMEM with 4.5% Glucose, supplemented with 10% FBS and 1× antibiotic-antimycotic. HepG2 and Hep3B cells were grown in MEM, supplemented with 10% FBS, 1× non-essential amino acids, 1× sodium pyruvate, and 1× antibiotic-antimycotic.

For in vitro enrichment of CSCs, cells were grown in a serum-free condition (SF) in DMEM/F12 (D6434, SIGMA), supplemented with 2 mM L-Glutamine, 20 ng/mL EGF (E9644, Sigma), and 10 ng/mL bFGF (F0291, Sigma), 1× antibiotic-antimycotic and 1% B-27 supplement (17504044, Life technologies).

### Self-renewal assay

Hepa1–6 cells were seeded in SF media at a density of 20,000 cells/well in a 6-well plate, and incubated for 4 days to allow the development of spheroids. After confirming formation of spheroids under the microscope, these spheroid were collected, treated with trypsin-EDTA for 4 min, and prepared in single-cell suspension with 500 cells/mL density with SF media. Seed single-cell suspension was placed at 2 μL/well in 96-well plate. The wells were marked with one or two cells. Additional 150 μL SF media was added in each marked well and monitor for 20 days. Images were taken daily to track and record progress.

### Hoechst-33342 efflux assay

After 7 days in culture, Hepa1–6 control or spheroid cells were harvested and 10^6^ cells/mL was prepared for each sample, centrifuged at 200 RCF for 5 min, washed 2 times and resuspended in 2% FBS/PBS. Hoechst-33342 was added to 5 μg/mL final concentration in 2% FBS/PBS and cells were incubated for 90 min at 37 °C, followed by 10 min on ice, followed by a single wash of ice cold 1X PBS. These cells were immediately mounted on glass slides and analyzed within 1 h on a fluorescence microscope with UV excitation settings. Cells with dye exclusion property were identified by overlapping UV filter images with bright field images.

### RNA interference (siRNA) and spheroid reversibility assay

For each siRNA used, we provided sense and antisense sequences in Additional file 1: Table S4. Lipofectamin RNAiMAX, and validated siRNA oligos targeting β-catenin (siCtnnb1: s63417, s63418; and siCTNNB1: s436, s437) or scrambled control (Cat # 4390849) were obtained (Lifetechnologies, CA, USA) and experiment was performed as per instructions provided. Knockdown validation of Ctnnb1 (mouse) and CTNNB1 (human) were confirmed by western blot (data provided in Additional file 1: Figure S3). We achieved > 70% knockdown efficiency by siRNA mediated transfection. Cells were seeded and allowed to grow spheroids for 3–4 days, transfected on day 4 with siRNAs, and incubated for 72 h (Hepa1–6) or 48 h (HepG2). For calculating CSCs, differentiated, and single cells, 5 random fields/well were selected and quantified. Data were normalized to untransfected control, followed by mean and S.D. calculation. Experiments were performed *n* = 3 times independently, and transfection was carried out using combination of two siRNAs targeting different exons (Additional file 1: Figure S3, Table S4).

### Animal experiments

All experimental procedures were approved by the Institutional Animal Care and Use Committee (IACUC) at the University of Louisville (UofL). All mice were housed in the UofL Research Resources Center (RRC) at 22 °C with 12-h light/dark cycles with free access to food and water. In vivo experiments using the Hepa1–6 cell line were performed in 12-week-old male C57L/J mice (Jackson laboratory, ME, USA). Each experimental group had *n* = 6 animals. For orthotopic inoculation, 2 × 10^6^ Hepa1–6 non-spheroid control cells or 7-day spheroids were injected into left liver lobe of an animal. Post injection, mice were monitored for 2 weeks and then euthanized on day 14 by carbon dioxide chamber procedure. Animal weight, liver weight, tumor weight, and tumor size were recorded for each animal.

### Statistical analysis

Data are presented as mean ± S.D. (*n* ≥ 3 per group). Comparisons were performed using Microsoft Excel-2013 suite by two-tail student’s t-test with equal variance (Redmond, USA). Results with *p* ≤ 0.05 were considered statistically significant.

## Results

Hepa1–6 was reported as a non-immunogenic murine cell line with an ability to form tumor in normal immunocompetent C57L/J mice, both orthotopically and subcutaneously [25–27], thus provide more clinically relevant mouse model to study tumorigenesis in presence of all confounding immune system components, contrary to athymic nude or SCID mouse models (lacks confounding immune system components). HepG2 and Hep3B secrete 17 major plasma proteins [28], and also express very high EpCAM levels, a well reported CSC marker in HCC [17]. To enrich and promote CSCs growth in HCC cell lines in vitro, we used serum-free culture method to maintain the undifferentiated status of the cells [29], which was widely adapted for CSC studies in various cancers [30, 31].

Unlike the previous studies in which CSC spheroids were enriched in low-attachment plates or mechanically induced [32], we used regular tissue culture-treated plates. All three cell lines (Hepa1–6, HepG2, and Hep3B) could successfully form spheroids in serum-free conditions (Additional file 1: Figure S1). Hepa1–6 and HepG2 cells could start losing adherence and form spheroids as little as 3–5 days, while the Hep3B cell, a p53 null cell line with mesenchymal phenotype and reported lung metastasis in nude mouse [28], took more than 25 days to develop spheroids. Numbers of spheroids generated in Hep3B cells were far less than Hepa1–6 and HepG2 cells.

### Identifying CSC like properties in Hepa1–6 HCC cells

**Fig. 1:**
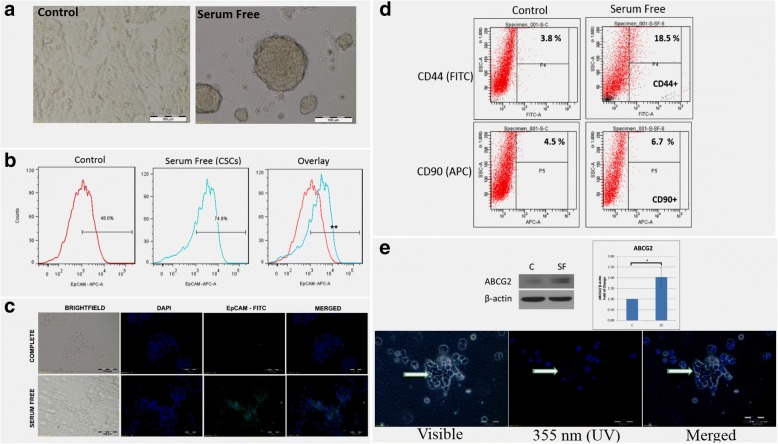
Identifying CSC like properties in Hepa1–6 spheroids: **a** Hepa1–6 adherent cells (left, control) and 7 days old spheroids (right, serum-free); **b** Flow cytometry data showing significant higher EpCAM expression in spheroids compared with control (Day 7, **p < 0.005, *n* = 4); **c** ICC staining for EpCAM (FITC - green) confirming flow cytometric findings that EpCAM upregulated in spheroids. Nuclear stained with DAPI (blue); **d** CSCs spheroids also showed significant increases in CD44 and CD90 compared with control (*p* < 0.05, *n* = 3). **e** Hoechst efflux assay: Hepa1–6 spheroids showed significantly higher ABCG2 expression by western blot (upper panel) and significantly increased Hoechst-33342 dye efflux activity (lower panel). SF = Hepa1–6 spheroids (*n* = 3 independent experiments)

**Fig. 2:**
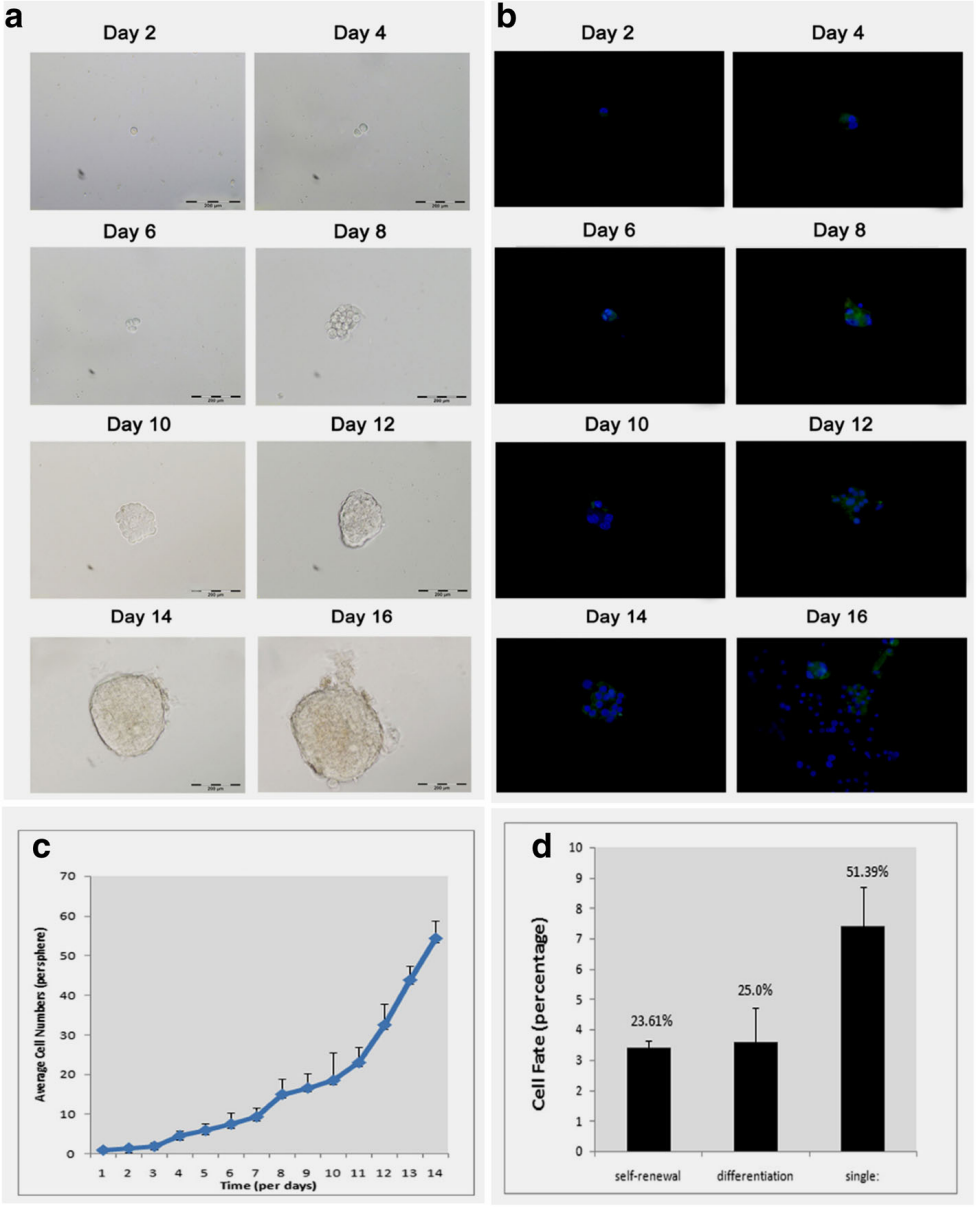
Self renewal assay for Hepa1–6 CSCs: Hepa1–6 CSC spheroids possess self-renewal capability in serum-free condition. Hepa1–6 cells were seeded in 96-well plate with appropriate dilution to achieve single cell/well, and observed for two weeks. **a** Representative bright field images of Hepa1–6 cells being self-renewed in serum free media at different time-points to form spheroids; **b** Fluorescence microscope images of growing spheroids at different time-points. DAPI (Blue) and FITC-EpCAM (CSC marker - Green) staining; **c** Bar graph showing data of Hepa1–6 cells being self-renewed to grow as spheroids; **d** Quantified cells for self-renewal (spheroid formation), differentiation (adherent growth), and single cell (quiescent cells). Images captured at 10× magnification (Bar = 200 μm)

In the first step, we had examined CSC like properties in HCC cells in vitro using established serum-free culture method [32]. Self-renewal capability is the fundamental characteristic of CSCs and the primary mechanism responsible for maintaining undifferentiated cells with CSC-like properties in tumors [33]. As shown in Fig. 1a, Hepa1–6 spheroids were induced in adherent tissue-culture treated plates using serum-free medium. These Hepa1–6 spheroids can serially pass for more than 10 generations in serum-free medium (data not shown, *n* = 2 times), an important property of self-renewal. We have confirmed and quantitated the self-renewal capacity of Hepa1–6 spheroids by minimal dilution assay using a 96-well plate and found that 24% of Hepa1–6 cells acquired self-renewal capability and form spheroids, while the rest were either in quiescence (51%) or non-spheroid differentiated stage (25%) (Fig. 2).

Since EpCAM is a well-accepted HCC-CSC marker, we first tested EpCAM expression in Hepa1–6 spheroids. The EpCAM expression was significantly increased in Hepa1–6 spheroids (74%, MFI 2660 ± 420) than non-sphere Hepa1–6 cell control (36%, MFI 723 ± 375; *p* < 0.001, *n* = 4) by flow cytometry (Fig. 1b), and was further confirmed by ICC staining (Fig. 1c). In addition, two more CSC surface markers, CD44 and CD90, were tested and both CD44 (15%; *p* < 0.05, *n* = 3) and CD90 (2%; *p* < 0.05, *n* = 3) were upregulated in the Hepa1–6 spheroids compared with non-sphere Hepa1–6 cell control (Fig. 1d). The increased CSC surface markers suggested that HCC-CSCs were enriched in the Hepa1–6 spheroids. To further confirm, the functional marker ABCG2 (an ATP-dependent, multidrug-resistant transporter) was investigated. Higher ABCG2 activity leads to higher drug efflux function, which can be indirectly measured by lower Hoechst-33342 staining. As showed in Fig. 1e, Hepa1–6 spheroids showed significantly higher ABCG2 protein expression, consistent with the lower Hoechst-33342 staining (higher ABCG2 efflux activity).

Taken together, spontaneously induced Hepa1–6 spheroids by serum-free culture exhibited CSC properties. Previous reports showed that both EpCAM and CD44 are targets of the Wnt/β-catenin pathway [17, 21]. Therefore, the components in Wnt/β-catenin signaling were further evaluated.

### Canonical Wnt/β-catenin pathway is upregulated in Hepa1–6 CSC spheroids

**Fig. 3:**
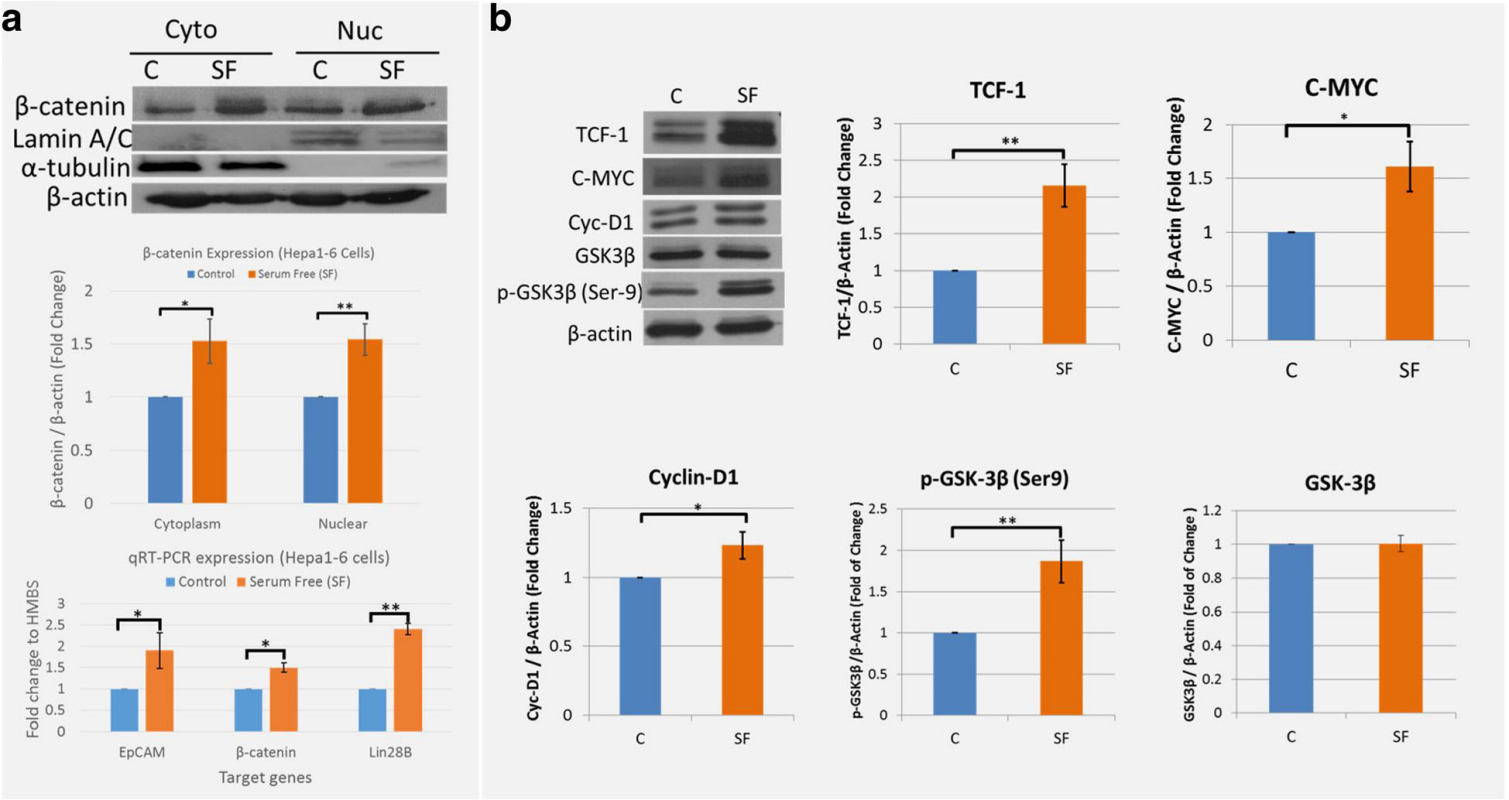
Expressions of β-catenin and Wnt components. Hepa1–6 cells were grown for 7 days in complete media (C) or serum-free media (SF); **a** Western blot analysis of cytoplasmic and nuclear β-catenin (upper panel), densitometry image analysis (middle panel), and qRT-PCR analysis of total RNA (lower panel); **b** Western blot analysis of total protein for downstream targets of canonical Wnt/β-catenin signaling and analysis of GSK-3β Ser-9 phosphorylation status. All blots from the same experiment, and normalized to β-actin. *(*p* ≤ 0.05), **(*p* ≤ 0.005), n = 3 independent experiments. Cyto = Cytoplasm; Nuc = Nuclear; C=Control; SF=Serum-free

The Wnt/β-catenin pathway is reported as a cardinal pathway for maintaining stem cells and highly implicated in HCC and CSCs [19, 34]. We investigated canonical Wnt/β-catenin signaling components by western blot and qRT-PCR. Our results indicated that Hepa1–6 spheroids showed increased β-catenin levels both in the cytoplasma (1.53 ± 0.21 fold; *p* ≤ 0.05, *n* = 3) and nucleus (1.54 ± 0.15 fold; *p* ≤ 0.001, *n* = 3) compared to control Hepa1–6 cells by western blot anlysis (Fig. 3a). We have also confirmed increased β-catenin levels in human HCC cell line - HepG2 spheroids (Additional file 1: Figure S2) which is consistent with findings in Hepa1–6 cells. Total RNA analysis by qRT-PCR further confirmed the increased β-catenin and stemness in Hepa1–6 spheroids – significant increase in both EpCAM and Lin28B (Fig. 3a). Downstream targets of canonical Wnt/β-catenin signaling, i.e., C-MYC, Cyclin-D1, LEF1, were proportionately upregulated with the increased β-catenin levels (Fig. 3b), suggesting that higher β-catenin levels in Hepa1–6 spheroids could affect downstream events. Hepa1–6 spheroids showed an increase in inactive GSK-3β levels (phosphorylated at Ser9) compared to control non-sphere Hepa1–6 cells (Fig. 3b), suggesting that GSK-3β activity at least partially contributed to increased β-catenin levels by protecting it from ubiquitin mediated degradation in Hepa1–6 spheroids where the Hepa1–6 CSCs could be maintained.

### Silencing β-catenin reverses spheroid phenotype of CSCs in EpCAM positive HCC cell lines

**Fig. 4:**
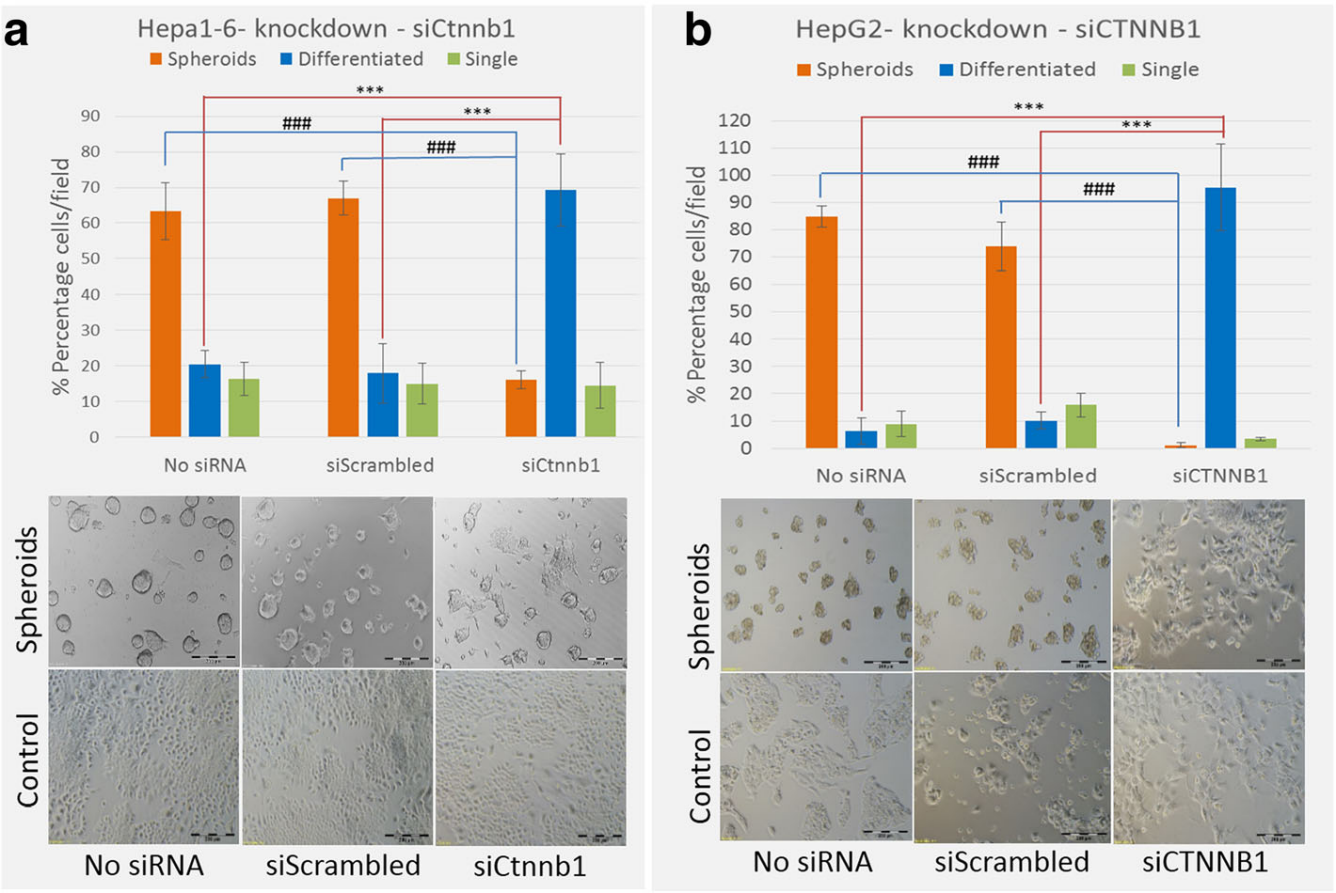
Silencing β-catenin reverses spheroid phenotype of CSCs: **a** Hepa1–6; **b** HepG2 cells were cultured as control or spheroids for 3 days, transient transfection with scrambled siRNA control or siCTNNB1, followed by 48 to 72 h incubation. Upper panels show average quantification (*n* = 5 random fields/group) of spheroids (orange), adherent (blue), and single cells (green). Error bar is representing SD. *n* = 3 independent experiments, *** (*p* ≤ 0.005, control), **###** (*p* ≤ 0.005, spheroids). Lower panel show visual representative images of corresponding experimental group

The upregulation of β-catenin led us to investigate the role of β-catenin in spontaneous CSC spheroid generation, and we knocked down Ctnnb1 (β-catenin gene) using siRNA to further study implication of loss of function of β-catenin in spontaneous spheroid formation. After 48–72 h of Ctnnb1 knockdown (*n* = 3 independent experiments), Hepa1–6 cells lost the spheroid phenotype and shifted to the differentiated phenotype, similar to their parent cells growing in the medium with serum (Fig. 4a). This shifted phenotype of HCC spheroid was further confirmed in HepG2, a human EpCAM positive cell line. Upon CTNNB1 knockdown by siRNA, HepG2 spheroids showed close to 98% reversal to differentiated phenotype in serum-free media (Fig. 4b). Loss of spheroid phenotype was far more effective in HepG2 compared with Hepa1–6 upon the siRNA-mediated knockdown of β-catenin.

### The induced HCC spheroids are more tumorigenic in vivo

**Fig. 5:**
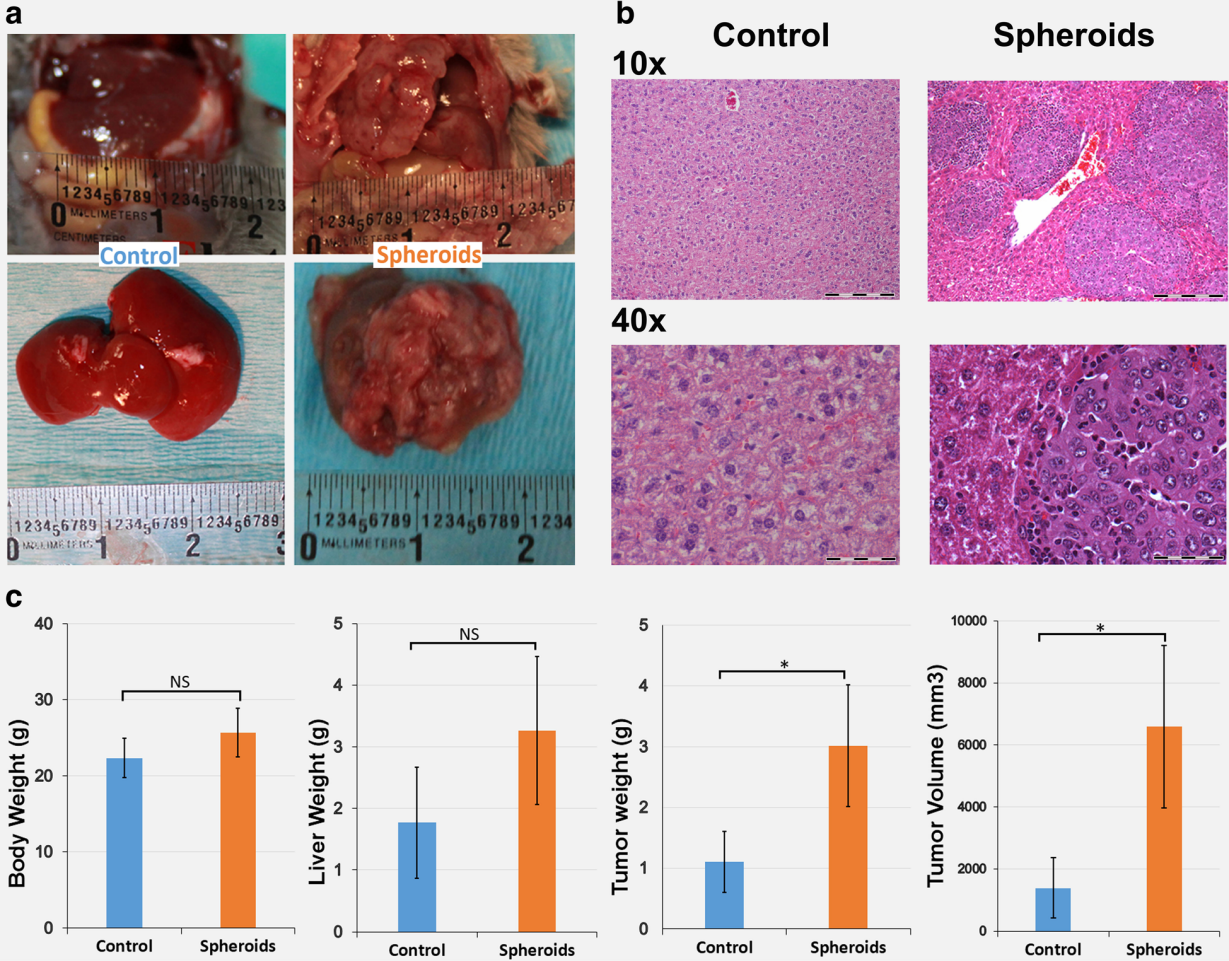
CSC spheroids possessed higher tumorigenic capability: 12 week old C57L/J mice were injected with 2 × 10^6^ control Hepa1–6 cells or Hepa1–6 spheroids (*n* = 6 mice/group) into left liver lobe. Tumors were allowed to grow for two weeks and mice were euthanized at the end of two weeks; **a** Representative liver tumors of control and spheroid group mice; **b** Representative H&E staining of control group and spheroid group, bright field images with 10× magnification (upper, bar = 200 μm) and 40× magnification (lower, bar = 50 μm); **c** Quantitative comparison of body weights, liver weights, tumor weights, and tumor volumes between control and spheroid group mice. *(*p* ≤ 0.05). NS (non-significant)

We used an orthotopic C57L/J mouse model to further test tumorigenic potential of Hepa1–6 spheroids where CSCs being enriched [27]. Hepa1–6 cell line possesses tumorigenic property in immunocompetent C57L/J mice [25, 26]. Seven-day cultured 2 × 10^6^ cells from Hepa1–6 spheroids as well as non-spheroid control cells were inoculated into upper left lobe of livers of 12-week-old immunocompetent C57L/J mice (*n* = 6 mice/group) and monitored for 2 weeks. Mice injected with Hepa1–6 spheroids developed more aggressive tumors with overall higher tumor volume (6593 ± 2615 mm^3^) compared with mice injected with control cells (1388 ± 968 mm^3^; *p* ≤ 0.05, *n* = 6) (Fig. 5c). Also, Hepa1–6 spheroids developed more aggressive tumors with significant higher tumor weight (3.1 ± 1 g) compared with mice injected with control cells (1.1 ± 0.5 g; *p* ≤ 0.05, *n* = 6) (Fig. 5c). The aggressive growth pattern of CSC tumors was also witnessed by multiple HCC nodules found at different lobe sites within the liver compared to control, suggesting that CSC spheroids possess increased tumor initiation and hepatic invasive capabilities (Fig. 5a). These findings were confirmed by H&E staining (Fig. 5b). Increased liver weight was also observed in the spheroid group (3.26 ± 1.2 g) compared with the control group (1.77 ± 0.9 g, NS, *n* = 6), which is likely due to aggressive growth and higher tumor mass in the spheroid injected group. This justifies the increased overall body weight by about 3 g in the spheroid group (25.68 ± 3.2 g) compared to control (22.35 ± 2.6 g; NS, *n* = 6) (Fig. 5c).

## Discussion

In this study, for the first time we showed that HCC CSC spheroids were induced using only serum-free media and adherent tissue-culture treated plates. These HCC spheroids showed significant increased expression of CSC surface markers and functional markers, indicating that CSCs were enriched in the HCC spheroids. Increased Wnt/β-catenin components were found in the HCC spheroids compared with control cells grown in the presence of serum. We also showed that β-catenin upregulation, at least partly, was GSK-3β dependent, caused transcription activities of the downstream targets including Cyc-D1, c-MYC, and LEF-1. Upon silencing β-catenin by siRNA, CSCs lost spheroid phenotype and shifted to differentiated phenotype even in serum-free media. The HCC orthotopic mouse model confirmed the higher in vivo tumorigenic potential of Hepa1–6 CSCs in immunocompetent liver microenvironment, for the first time.

Serum-free conditions plus growth factors allow cells to grow in an anchorage-independent manner, and is a well-documented standard method for maintaining undifferentiated cells [29, 35–37]. Spheroids formed in serum-free culture have been suggested to mimic not only the phenotype but also the genotype of the primary tumor [31]. In fact, serum-free culture has been widely accepted and used for in vitro enrichment of CSCs [32]. A recent study reported that simply generating multicellular spheres followed by reversal to attached cell line can significantly change the cell phenotype, which can be used as in vitro metastatic model [35]. Therefore, spheroid cultures provide a useful approach to enrich CSCs and to study CSC-like cells present in primary tumors with the added advantage of reproducibility and validity of findings. One study previously reported that not all cell lines can enrich CSC-like cells in spheroid culture, and each cancer cell line should be evaluated carefully and subjectively [38]. We successfully induced HCC spheroids in both human and mouse cell lines in vitro and these induced HCC spheroids have shown the characteristics of CSCs. Our current study further advocates the use of a serum-free technique to enrich HCC-CSCs. For the first time, without using ultra-low attachment plates, we showed that HCC cells could generate spheroids in an adherent culture environment in normal tissue culture plates, and these CSCs spheroids showed upregulated β-catenin, the key component of canonical Wnt pathway. Upon silencing β-catenin, the HCC spheroids switch back to a differentiated phenotype in serum-free culture environment, suggesting an important role of the canonical Wnt pathway in HCC-CSC activation and in the “differentiation to dedifferentiation” switch. Further study is needed in this direction to elucidate detailed mechanism.

The existence CSCs is no longer debatable area since definitive evidence recently identified CSCs by three independent lineage tracking studies [39–41]. However, the origin of CSCs is not yet elucidated and is still an active area of investigation [42, 43]. Primarily two arguments exist:(1) CSCs originate by dedifferentiation from either cancer cells or normal cells, and (2) CSCs originate by mutations and biochemical changes in a tissue-specific pool of normal progenitor or stem-cell compartment [44–48]. A study by Yamanaka et al. showed that terminally differentiated fibroblast cells possess reprogramming capability to go back to the pluripotent stem cell niche [49]. Considering unpredictable tumor cell fate and the poorly understood tumor microenvironment, the origin of CSCs from differentiated cancer cells is a likely possibility [50, 51]. The data we provide in this study show that β-catenin plays a strategic role in switching from a differentiated cancer cell phenotype to a CSC phenotype. This observation strongly implies that the heterogeneous cancer cells in a tumor mass could be an important pool of cancer-cell progenitors. Our speculation is supported by the evident increase in both surface and functional markers, as well as the tumor-initiating ability of HCC cell spheroids in immunocompetent liver microenvironment of mouse model. Further study is encouraged to investigate specific biomarkers in order to identify human HCC-CSCs, which could then be used in patients to evaluate potential treatment, response and prognosis.

## Conclusion

In conclusion, successfully induced Hepa1–6 spheroids were identified with CSC-like properties. Aberrant β-catenin upregulation mediated by GSK-3β was observed in the Hepa1–6 spheroids. The β-catenin mediated CSC enrichment in the induced spheroids possesses the capability of tumor initiation in immunocompetent mice. Without genetic manipulation, our study suggests plausible cell dedifferentiation mediated by β-catenin contributes to CSC-initiated tumor growth in vivo in HCC.

## Additional file

Additional file 1: Supplementary experiments and routine methods. **Figure S1.** Spheroid formation capability of HCC cell lines. **Figure S2.** β-catenin expression in HepG2 cell line. **Figure S3.** Knockdown efficiency of siRNA transfection. Routine Methods: Immunocytochemistry (ICC) Staining, Flow-Cytometry Analysis, Protein Extraction and Western Blot. **Table S1**. Primary antibodies (catalog info and dilution). **Table S2.** HRP conjugated secondary antibodies (catalog info and dilution). **Table S3.** Important reagents (catalog info). **Table S4**. Sequence details for RNA interference experiments. (DOCX 573 kb)

## Abbreviations

ABCG2: TP Binding Cassette Subfamily G Member 2
c-Myc: V-Myc Avian Myelocytomatosis Viral Oncogene Homolog
CSCs: Cancer stem cells; Cancer stem-like cells; Tumor initiating cells
CTNNB1: Catenin (Cadherin-Associated Protein), Beta 1 (beta-catenin)
Cyc-D1: Cyclin-D1
EpCAM: Epithelial cell adhesion molecule
GSK3β: Glycogen synthase kinase 3 beta
p-GSK3β: Serine-9 phosphorylated GSK3β
SF: Serum-free media condition
TCF-1: Transcription factor 7, T-cell specific, TCF7
