# Supplementary material for: Enrichment of cancer stem cells via β-catenin contributing to the tumorigenesis of hepatocellular carcinoma
Source: BMC Cancer. 2018 Aug 3;18:783. doi: 10.1186/s12885-018-4683-0 (PMC6091111; doi:10.1186/s12885-018-4683-0)
Supplement: Supplementary file 1 — Supplementary experiments and routine methods. Figure S1. Spheroid formation capability of HCC cell lines. Figure S2. β-catenin expression in HepG2 cell line. Figure S3. Knockdown efficiency of siRNA transfection. Routine Methods: Immunocytochemistry (ICC) Staining, Flow-Cytometry Analysis, Protein Extraction and Western Blot. Table S1. Primary antibodies (catalog info and dilution). Table S2. HRP conjugated secondary antibodies (catalog info and dilution). Table S3. Important reagents (catalog info). Table S4. Sequence details for RNA interference experiments. (DOCX 573 kb) [file 12885_2018_4683_MOESM1_ESM.docx]

**ADDITIONAL FILE -1**

Supplementary experiments and routine methods.

**Figure-S1:** Spheroid formation capability of HCC cell lines - HCC cell lines can form CSC spheroids in adherent tissue culture plates using serum-free culture media.


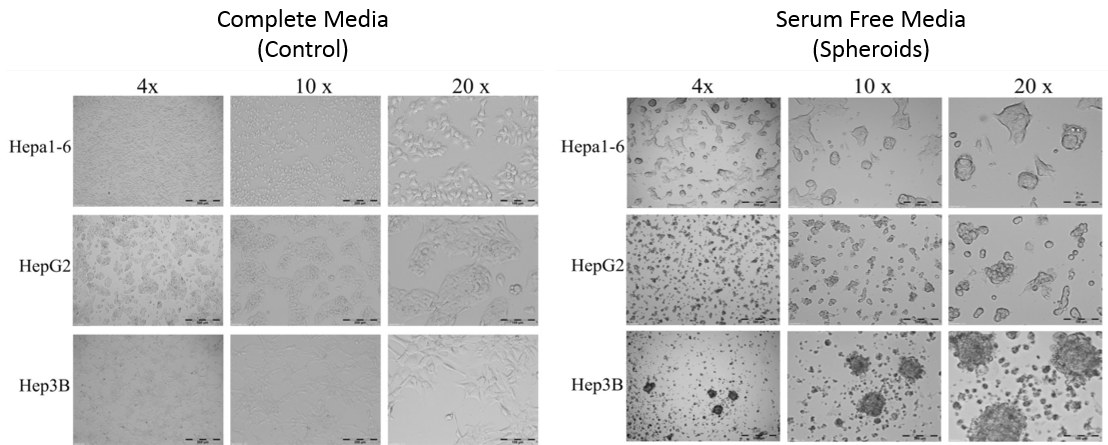


**Figure-S1.** **Spheroid formation capability of HCC cell lines:** All three HCC cell lines possesses capability to generate spheroids in serum-free culture media. Images representing control group (left panel) were normal HCC corresponding cell lines growing in complete media in T-75 tissue culture flasks. Images representing spheroids (right panel) were taken at day-7 for Hepa1-6 and HepG2, and day-30 for Hep3B. Light microscope images. Numbers i.e. 4x, 10x, 20x, representing fold magnification of images.

**Figure-S2:** Expression of β-catenin in HepG2 cell line – control v/s spheroids.

**
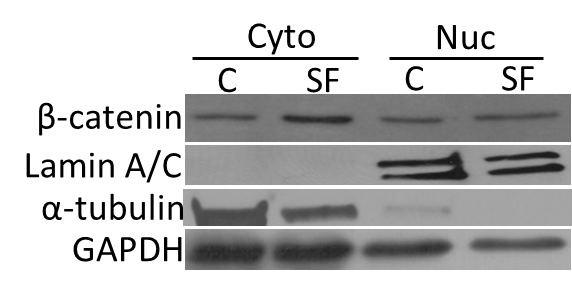

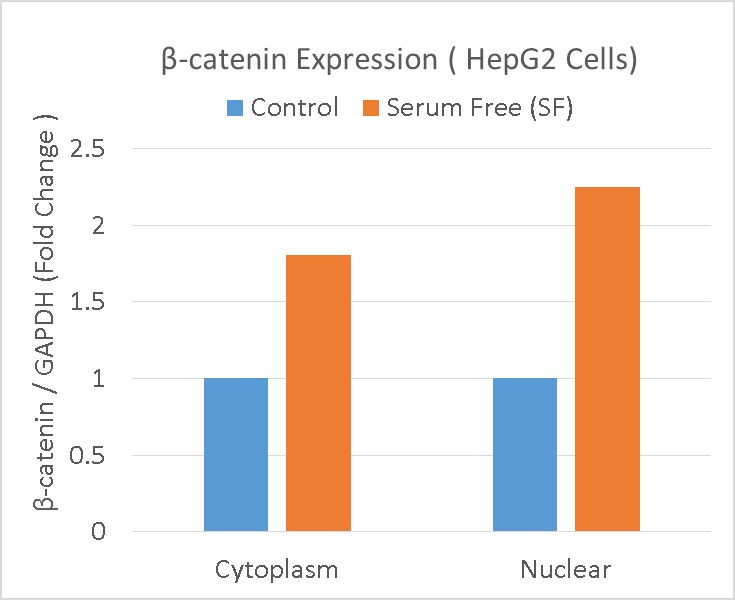
**

**Figure-S2. Expression of β-catenin in HepG2 cell line:** Both cytoplasmic and nuclear β-catenin levels were increased in day-7 HepG2 spheroids compared with control. Representing western blot and corresponding densitometry analysis showed. Lamin A/C and α-tubulin used as control for nuclear and cytoplasmic fractions respectively.

**Figure-S3:** Knockdown efficiency of siRNA transfection.

**
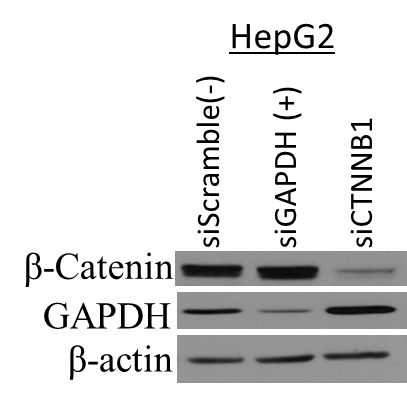

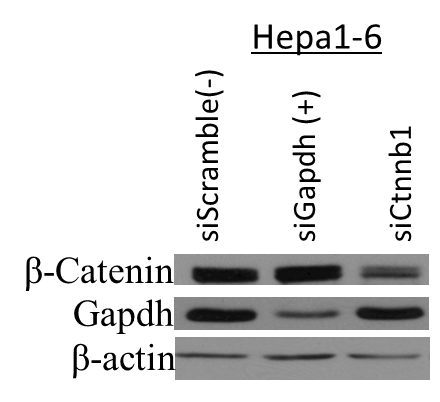
**

**Figure-S3. Transient knockdown of β-catenin by siRNA:** Mixture of 2 different siRNAs (25 pmol each) targeting separate axon sequences of Ctnnb1 mRNA (CTNNB1 for HepG2) were used to knockdown β-catenin. Using GAPDH as positive control during optimization, we have achieved consistent >70% knockdown efficacy in both Hepa1-6 and HepG2 cell lines (n=3 independent experiments). Sequences for siRNA are provided in supplementary table – S4.

**Routine Methods:**

**Immunocytochemistry (ICC) Staining:** Hepa1-6 control or spheroid cells were grown in 8 well Nunc™ Lab-Tek-II chamber slide (154534, Thermo scientific) for 7 days (seeded at 100 cells/well to avoid overgrowth at the end of 7 days). Media was changed on the 4th day to ensure viability. Cells were fixed with 4% PFA for 30 minutes. Fixed cells were washed 2 times for 15 minutes with PBS-T (0.05% tween in PBS with Ca++ and Mg++), and blocked with 10% BSA in PBS for 20 minutes. Blocked cells were washed again for 2 times for 15 minutes with PBS-T and incubated with 100 µL primary anti-EpCAM FITC conjugated antibody (Clone G8.8, anti-mouse CD326, eBiosciences, USA) at room temperature for 2 hours (1:100 dilution in 2% FBS in PBS-T). Cells were washed 3 times for 10 minute each by PBS-T, counter stained for nuclear staining with DAPI, followed by 2 times 10 minute PBS-T washes. Plastic chambers were removed and slide surface with cells were sealed with coverslip using Vectashield hard-bound (H-1500, Vector Lab, Burlingame, CA). The slides were examined using Olympus 1×51 microscope at 20x magnification using the Olympus DP72 digital camera via the cellSens Dimension imaging system (Olympus, Pittsburgh, PA) with FITC, DAPI and bright field settings. Digital images were taken, stored and then overlapped using software tool to analyzed for FITC positive cells in each experiments.

**Flow-Cytometry Analysis:** Hepa1-6 control or 7-day spheroid cells grown in 100 mm tissue-culture treated discs were collected by trypsin treatment followed by gentle cell scraping, centrifuged at 300 RCF to remove media and resuspended in an equal volume of ice-cold 2% BSA/PBS. Single cell suspension was prepared by repeated pipetting for 20-30 times followed by passing the cells through 40-micron strainer to remove cell clumps. Using a hemocytometer, cell numbers were determined and single-cell suspension was verified. Samples were then centrifuged for 5 minutes at 300 RCF to obtain cell pellets, and resuspended in appropriate volume of 2% BSA/PBS to obtain 10^6^ cells/100 µL. Corresponding unstained and single-stained controls were separated for each sample. Primary antibodies were added in 100 µL of cell suspension as per datasheet or in-house optimized dilution (1:100 for most antibodies). Samples were gently vortexed and incubated in the dark on ice for 1 hour. After incubation, cells were washed once with 1 mL 2%BSA/PBS, resuspended in 500 µL 2% BSA/PBS, transferred into labeled flow-tubes, and analyzed. Data were recorded on BD FACSCanto and data analysis was performed by FlowJo software suite.

**Protein Extraction and Western Blot:** To extract total protein, adherent cells were washed with 1X PBS and lysed by adding SDS lysis buffer supplemented with protease and phosphatase inhibitors (78443, Thermo scientific, USA), directly into culture wells (100 µL per well of 6-well plate or 500 µL per 100 mm plate). Spheroids cells were collected by gentle scraping, transferred to a 15 mL tube, centrifuged for 5 minutes at 200 RCF to collect cells followed by 1x PBS washes to remove media traces. Lysis buffer was then added (100 µL lysis buffer per 20 µL packed cell volume). After adding lysis buffer, lysates were transferred to 1.5 mL microfuge tubes and sonicated for 5 seconds on ice. After 30 minutes incubation on ice, lysates were centrifuged for 30 minutes at 14,000 RCF at 4 °C. Supernatant containing total cellular protein was collected carefully and transferred to a new labeled 1.5 mL microfuge tube. Purified protein samples were stored at -80 °C until further use. For cytoplasmic and nuclear protein extraction, we used a commercial fractionation kit (78833, Thermofisher Scientific, USA). Fractionation was performed as per the manufacturer’s protocol. Lamin A/C and α-tubulin used as control for nuclear and cytoplasmic fractions respectively.Extracted protein was stored at -80 °C until further use.

All antibody source, catalog information, and dilutions are provided in supplementary table S1 and table S2. Extracted proteins were quantified using the Bradford assay as per the manufacturer’s protocol (500-0006, Biorad, USA) in a 96-well plate format using the ELISA plate reader (MultiSKAN MCC/340, Thermofisher Scientific). Equal protein loading was performed (20 µg each, SDS reduced boiled samples) using reducing SDS-PAGE electrophoresis on 8% gel. Protein was then electro transferred to 0.2 micron PVDF membrane (Amersham, GE). Membrane was blocked in 5% non-fat milk for 1 hour at room temperature. The membrane was then incubated overnight in the primary antibody at an optimized working concentration. Next day, the membrane was washed 3 times with TBST for 7 minutes each, and was incubated for 1 hour in respective secondary HRP conjugated antibody. Secondary antibody blot was washed 3 times by TBST, followed by 1 wash of TBS, and then incubated in darkness for 1 minute in Western Blot Luminol Reagent (Santacruz Biotech, Cat # sc-2048). Chemiluminescence signals were detected in the darkroom using x-ray films. Densitometry analyses were conducted using ImageJ software (NIH, Bethesda, USA).

**Antibodies used in western blot assay.**  Secondary antibody was kept consistent with source of primary antibodies in each experiment, i.e. if primary antibody was from cell signaling – secondary antibody was also from the cell signaling in that specific experiment - consistent with datasheet experimental conditions provided by commercial vendor(s). EMD Millipore primary antibodies were detected by secondary antibodies from cell signaling.

**Table –S1:** Primary antibodies (catalog info and dilution)

| **Target** | **Source** | **Catalog** | **Dilution / assay** |
| --- | --- | --- | --- |
| β-Catenin | SantaCruz Biotech | sc-7963 | 1:2000 / Western blot |
| β-actin | SantaCruz Biotech | sc-81178 | 1:2000 / Western blot |
| GAPDH | SantaCruz Biotech | sc-365062 | 1:1000 / Western blot |
| GSK3β | Cell Signaling | 9832 | 1:2000 / Western blot |
| phospho-GSK3β (Ser-9) | Cell Signaling | 5558 | 1:2000 / Western blot |
| ABCG2 | Cell Signaling | 4477 | 1:1000 / Western blot |
| Cyclin-D1 | Cell Signaling | 2978 | 1:2000 / Western blot |
| TCF-1 | Cell Signaling | 2203 | 1:1000 / Western blot |
| C-MYC | Cell Signaling | 5605 | 1:1000 / Western blot |
| Lamin A/C | EMD Millipore | 05-714 | 1:500 / Western blot |
| α-tubulin | EMD Millipore | 04-1117 | 1:5000 / Western blot |

**Table –S2:** HRP conjugated secondary antibodies (catalog info and dilution)

| **Target** | **Source** | **Catalog** | **Dilution / assay** |
| --- | --- | --- | --- |
| Anti-Mouse IgG | Cell signaling | 7076 | 1:4000 / Western blot |
| Anti-Mouse IgG | SantaCruz Biotech | sc-2005 | 1:4000 / Western blot |
| Anti-Rabbit IgG | Cell Signaling | 7074 | 1:5000 / Western blot |
| Anti-Rabbit IgG | SantaCruz Biotech | sc-2004 | 1:5000 / Western blot |

**Table –S3:** Important reagents (catalog info)

| **Name** | **Source** | **Catalog** |
| --- | --- | --- |
| Hepa1-6 | ATCC | CRL-1830 |
| Hep3B | ATCC | HB-8064 |
| HepG2 | ATCC | HepG2 |
| DMEM with 4.5% Glucose | Corning | 10-013-CV |
| FBS | SIGMA | F2442 |
| Antibiotic-antimycotic | Corning | 30-004-CI |
| MEM | GIBCO | 11095-080 |
| Non-essential amino acids (100x) | GIBCO | 11095-080 |
| Sodium pyruvate (100x) | GIBCO | 11360-070 |
| Lipofectamin RNAiMAX | Life Technologies | 13778075 |
| Hoechst-33342 | Life Technologies | H1399 |
| DMEM/F12 (1:1) | SIGMA | D6434 |
| L-Glutamine | SIGMA | G7513 |

**Table –S4:** Sequence details for RNA interference experiments

| **siRNA used (Lifetech #)** | **PubChem SID** | **Sequence (5’ -> 3’)** | **Length** |
| --- | --- | --- | --- |
| siCtnnb1 (s63417) | N/A | Sense: CACUUGCAAUAAUUACAAAtt  Antisense: UUUGUAAUUAUUGCAAGUGag | 21  21 |
| siCtnnb1 (s63418) | N/A | Sense: GACUCAAUACCAUUCCAUUtt  Antisense: AAUGGAAUGGUAUUGAGUCct | 21  21 |
| siCTNNB1 (s436) | 160757058 | Sense: GGACCUAUACUUACGAAAAtt  Antisense: UUUUCGUAAGUAUAGGUCCtc | 21  21 |
| siCTNNB1 (s437) | 160757163 | Sense: GGAUGUUCACAACCGAAUUtt  Antisense: AAUUCGGUUGUGAACAUCCcg | 21  21 |
